# Supplementary material for: Xanthine Oxidase Inhibition and Anti-LDL Oxidation by Prenylated Isoflavones from Flemingia philippinensis Root
Source: Molecules. 2020 Jul 6;25(13):3074. doi: 10.3390/molecules25133074 (PMC7411605; doi:10.3390/molecules25133074)
Supplement: Supplementary file 1 [file molecules-25-03074-s001.pdf]

Supplementary materials

Xanthine Oxidase Inhibition and Anti-LDL Oxidation by Prenylated Isoflavones from *Flemingia philippinensis* root

Jeong Yoon Kim[1], Yan Wang[2], Zuo Peng Li[1], Aizhamal Baiseitova[1], Yeong Jun Ban[1], Ki Hun Park[1,*]

[a]*Division of Applied Life Science (BK21 plus), IALS, Gyeongsang National University, Jinju, 52828, Republic of Korea*

[b]*College of Food and Biological Engineering, Qiqihar University, Qiqihar 161006, China*

► **Contents**

Figure S1-S20: Structural information through 1D and 2D NMR of nine isoflavones (**1**-**9**)

Figure S21-S23: Enzyme kinetics of isoflavones (**1**-**9**)

Figure S24 and Table S1: Results of fluorescence quenching between XO and isoflavones

Figure S25: Effects of isoflavones (**2**-**5**) by measuring ApoB fragmentation at 1 and 2 μM

Table S2: UPLC-Q-TOF/MS analysis of isoflavones from *F. philippienesis*

Figure S26: Amino acids sequence of xanthine oxidase from bovine milk

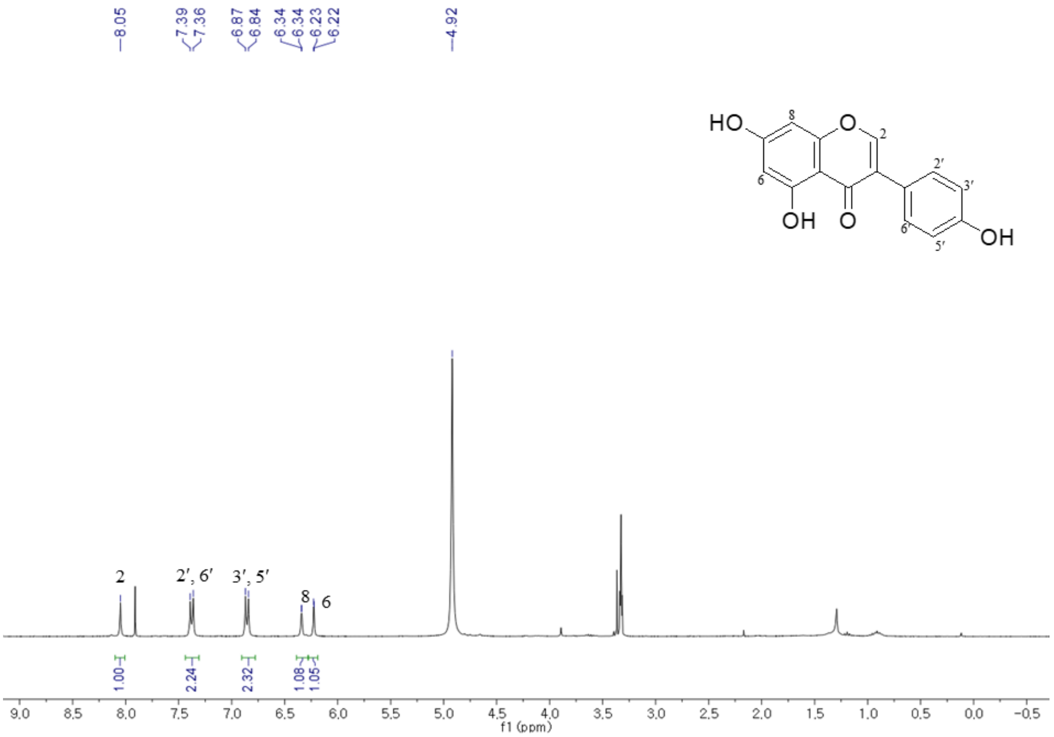

Figure S1. ^{1}H-NMR spectrum of compound **1** (500 MHz, CD_3OD)

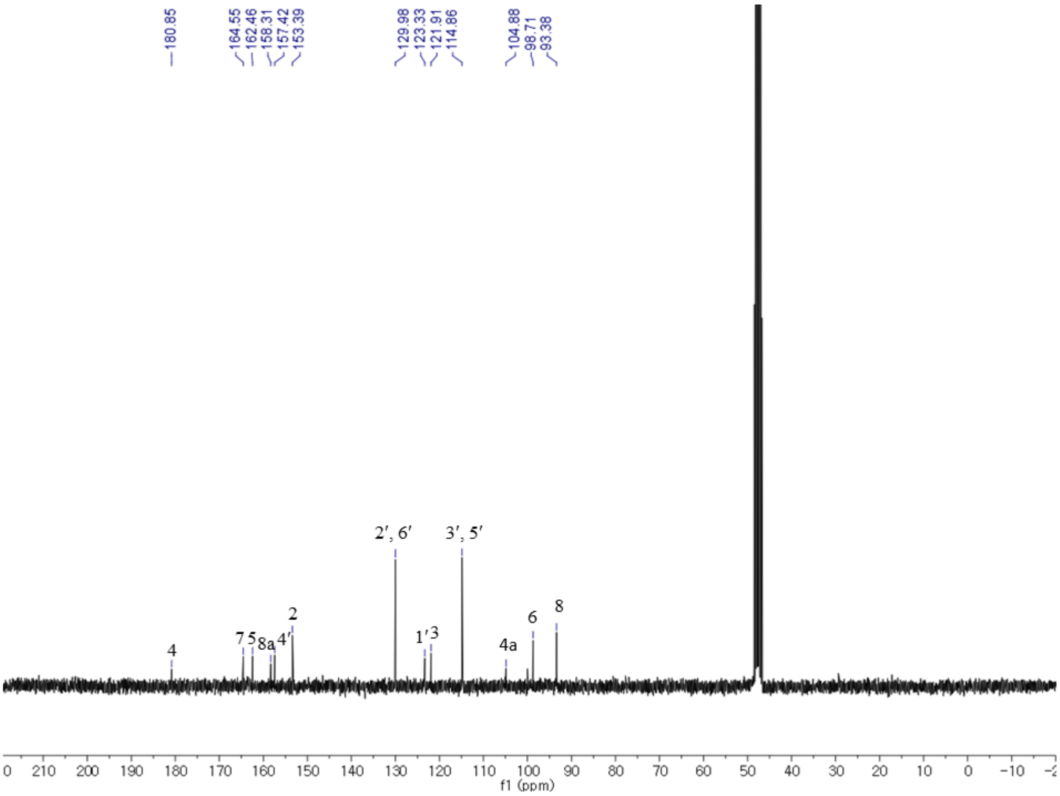

Figure S2. ^{13}C-NMR spectrum of compound **1** (125 MHz, CD_3OD)

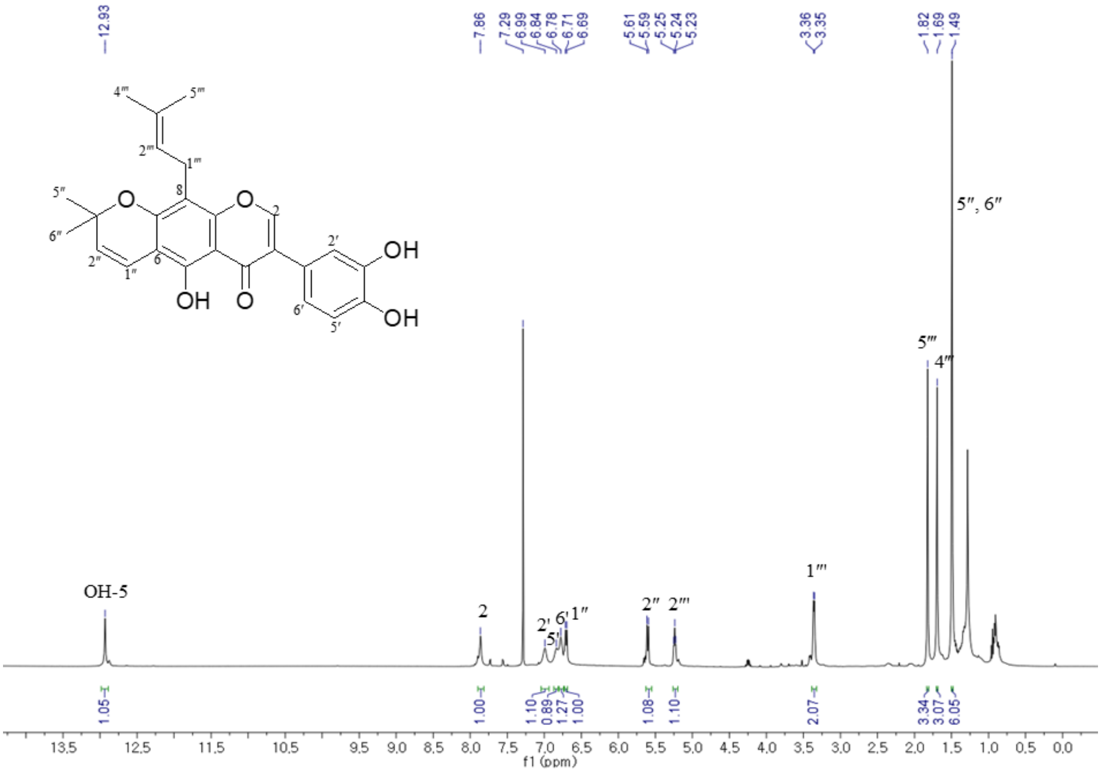

Figure S3. ^{1}H-NMR spectrum of compound **2** (500 MHz, $CDCl_3$)

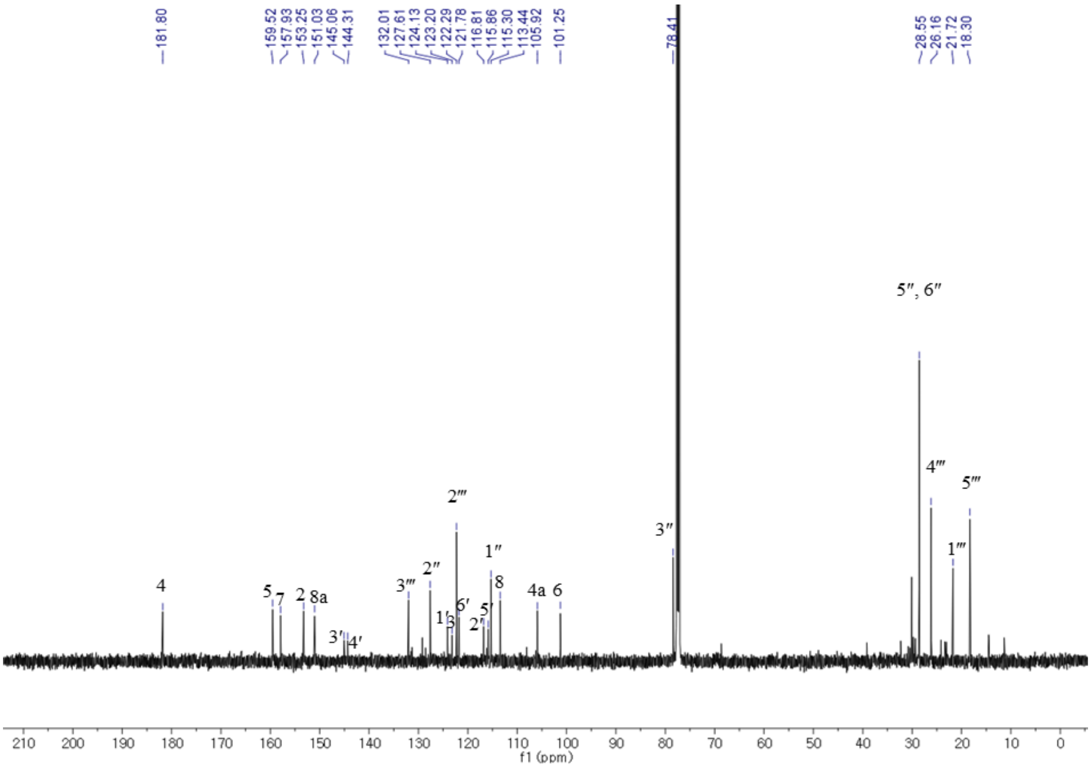

Figure S4. ^{13}C-NMR spectrum of compound **2** (125 MHz, $CDCl_3$)

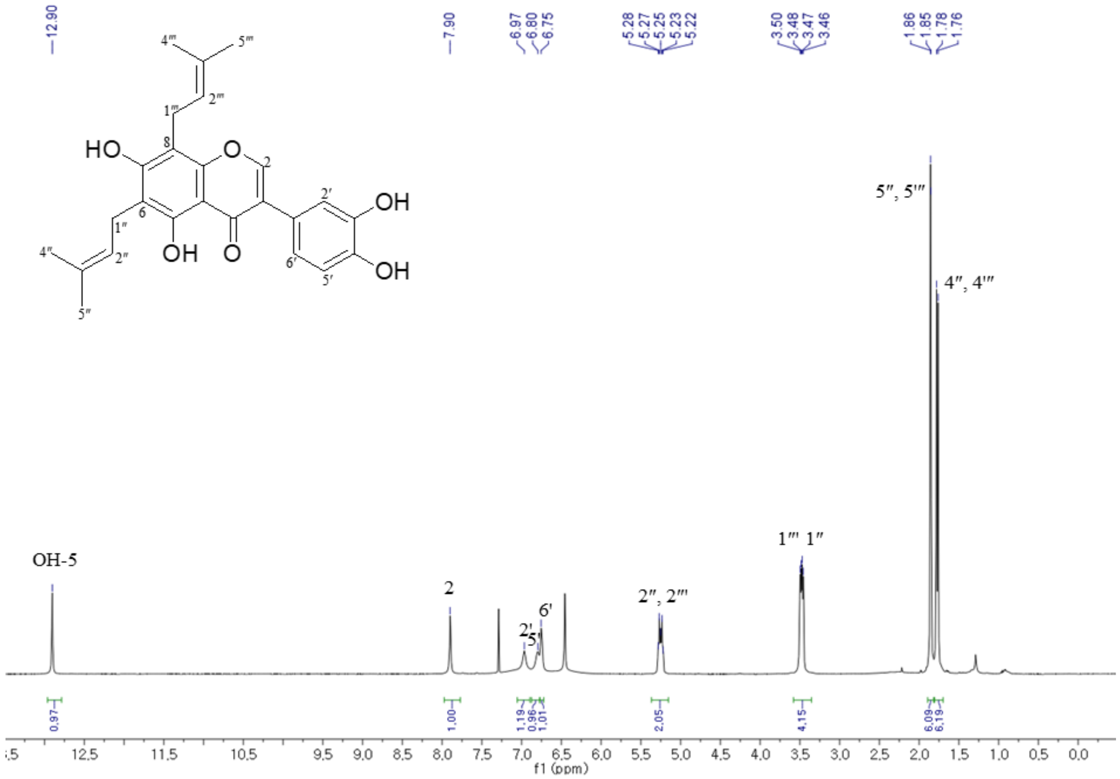

Figure S5. ^{1}H-NMR spectrum of compound **3** (500 MHz, Acetone-d_6)

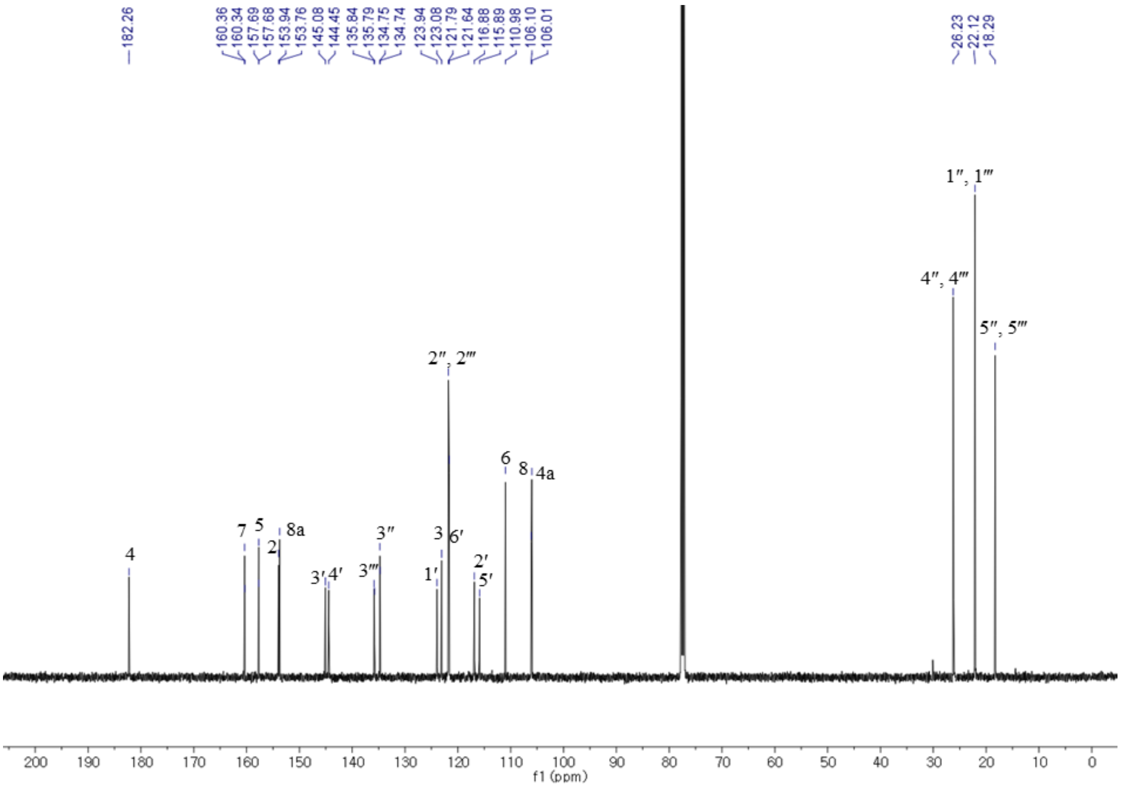

Figure S6. ^{13}C-NMR spectrum of compound **3** (125 MHz, Acetone-d_6)

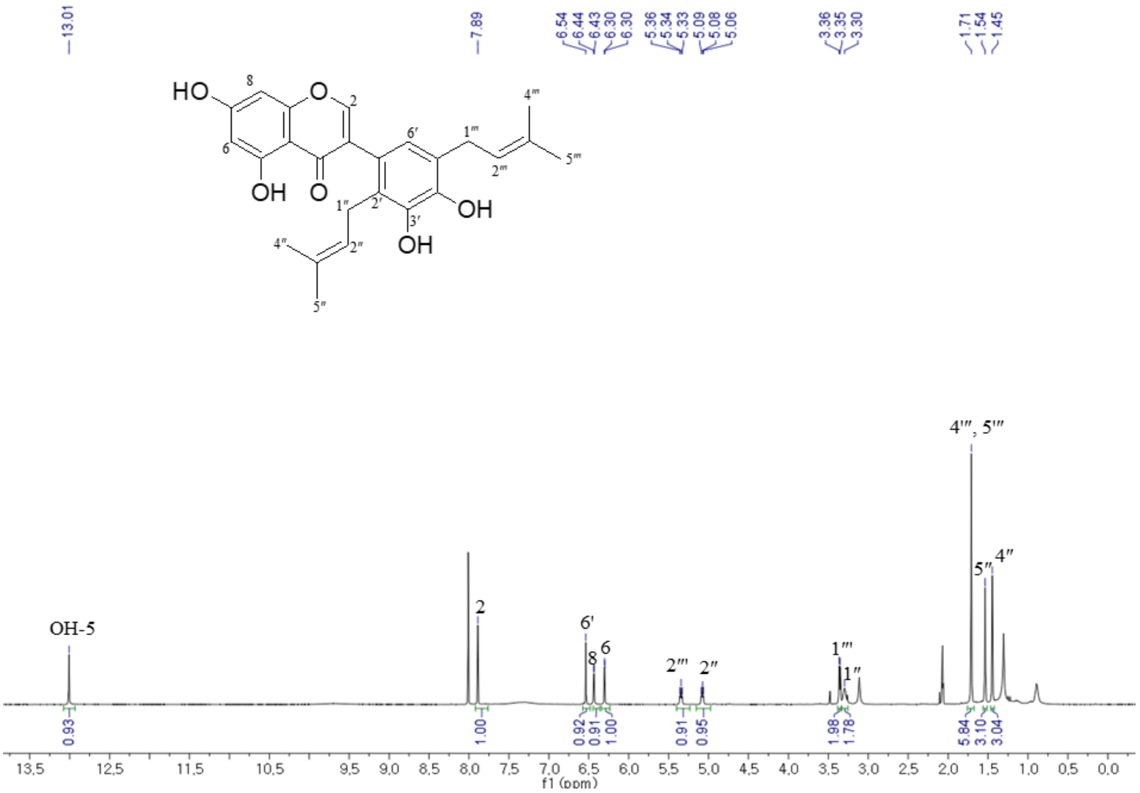

Figure S7. ^{1}H-NMR spectrum of compound **4** (500 MHz, Acetone-d_6)

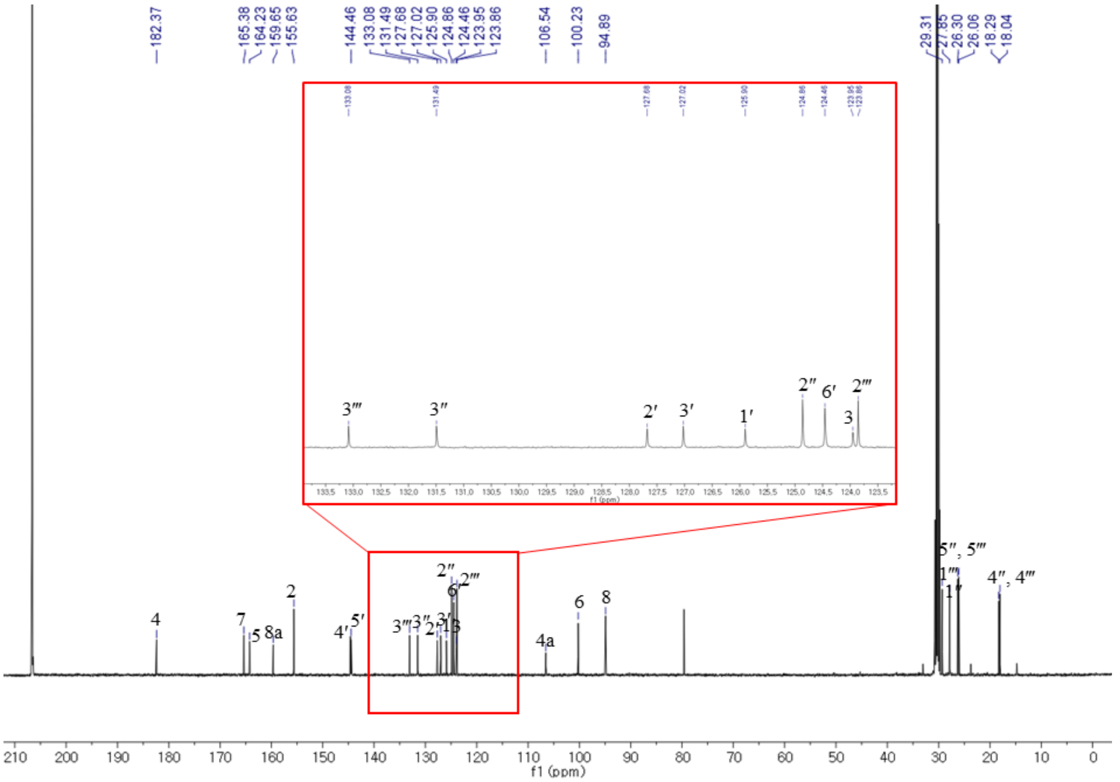

Figure S8. ^{13}C-NMR spectrum of compound **4** (125 MHz, Acetone-d_6)

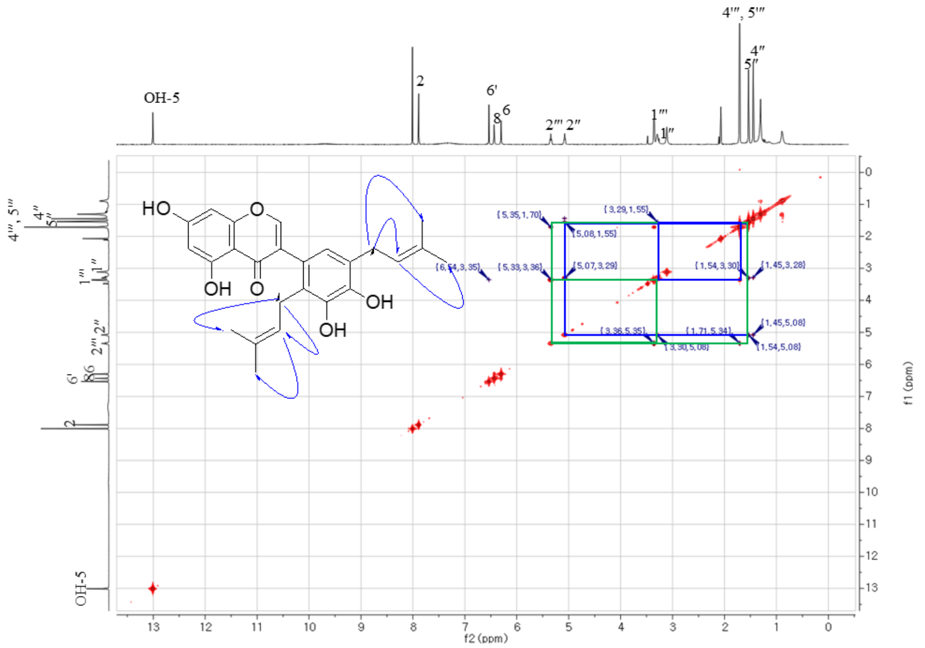

Figure S9. COSY spectrum of compound **4** (500 MHz, Acetone-d_6)

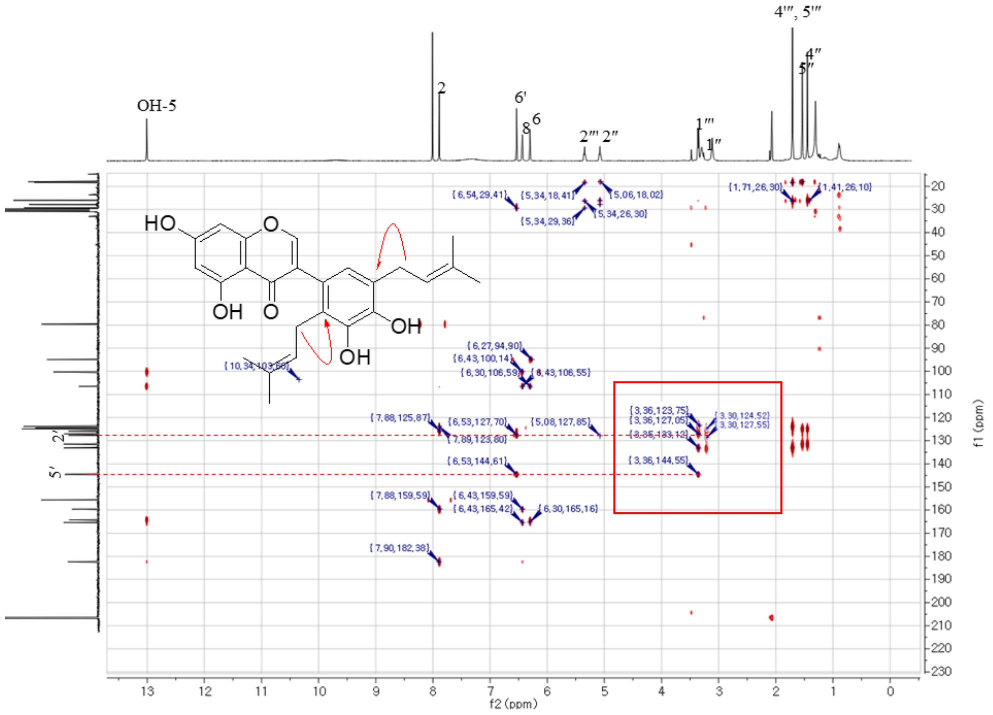

Figure S10. HMBC spectrum of compound **4** (500 MHz, Acetone-d_6)

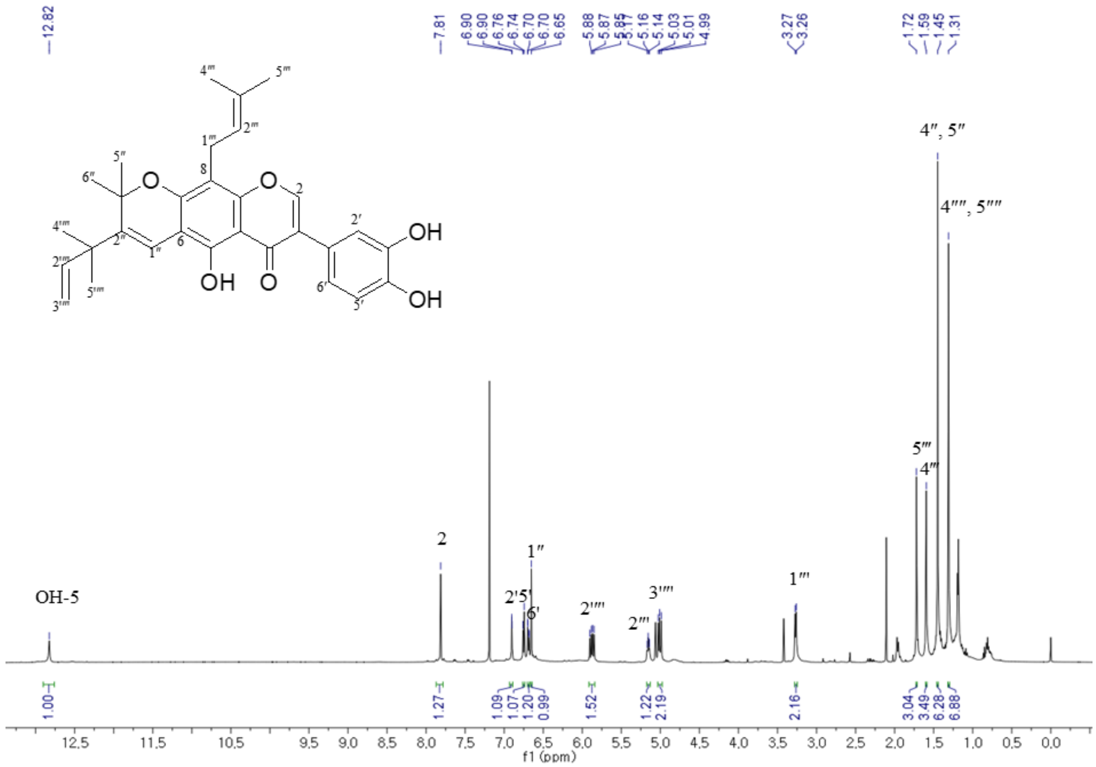

Figure S11. ^{1}H-NMR spectrum of compound **5** (500 MHz, $CDCl_3$)

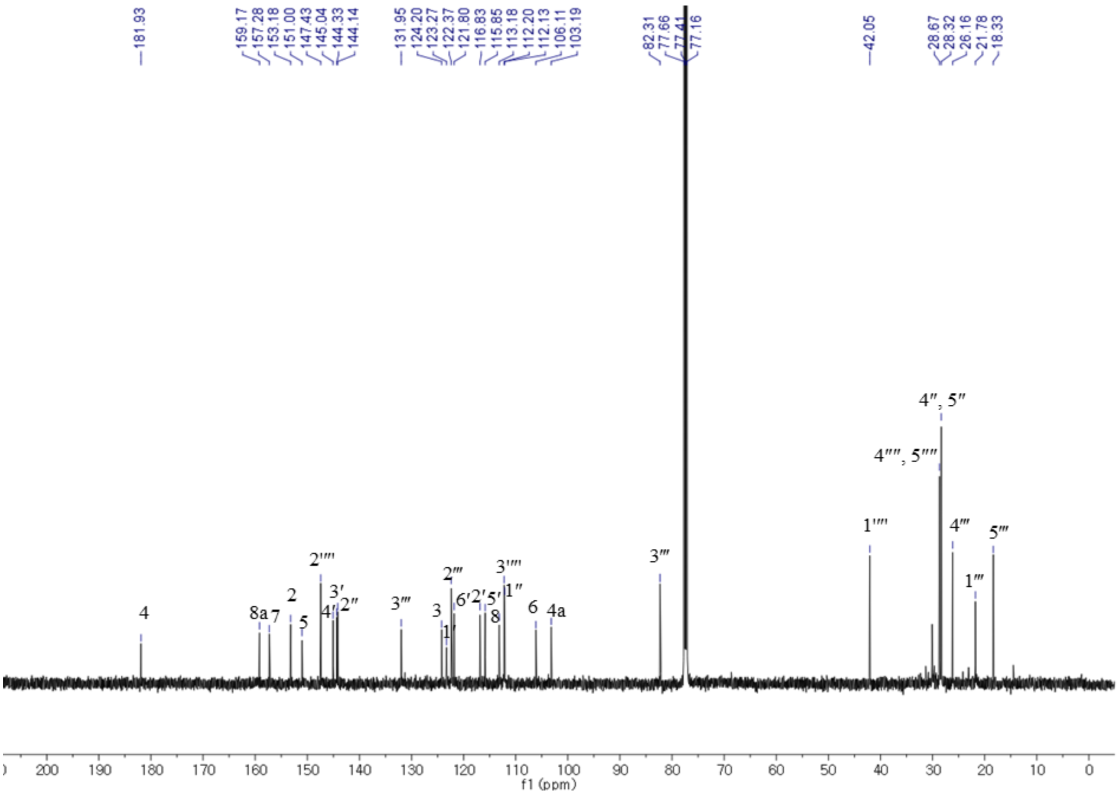

Figure S12. ^{13}C-NMR spectrum of compound **5** (125 MHz, $CDCl_3$)

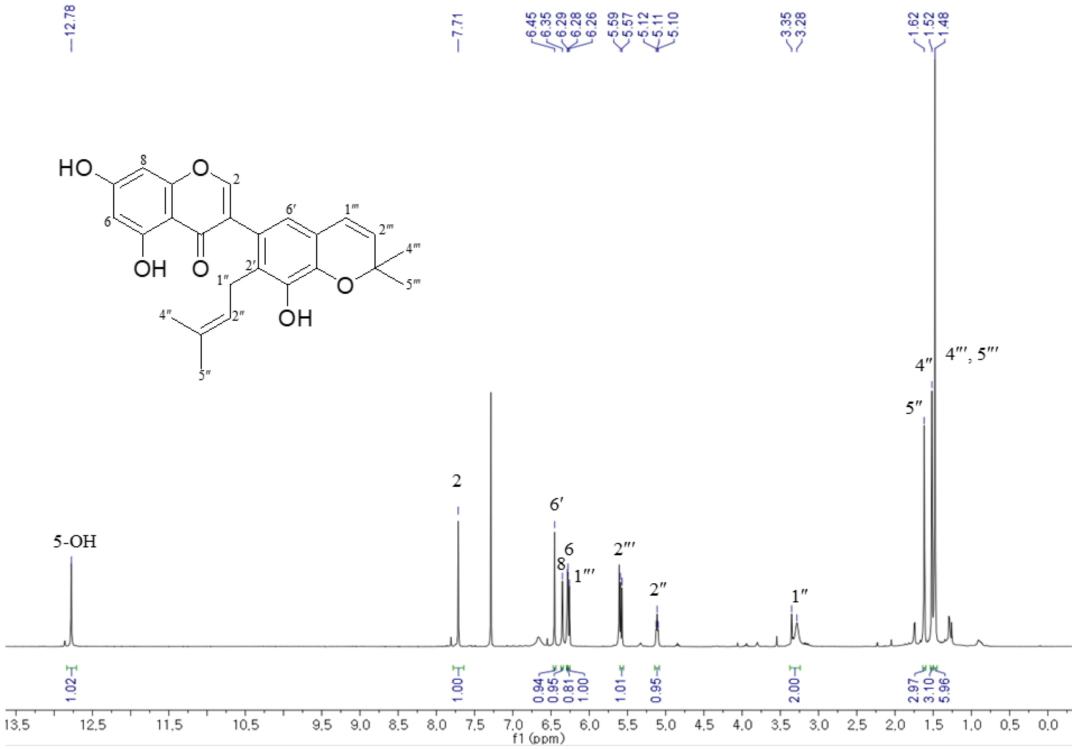

Figure S13. ^{1}H-NMR spectrum of compound **6** (500 MHz, $CDCl_3$)

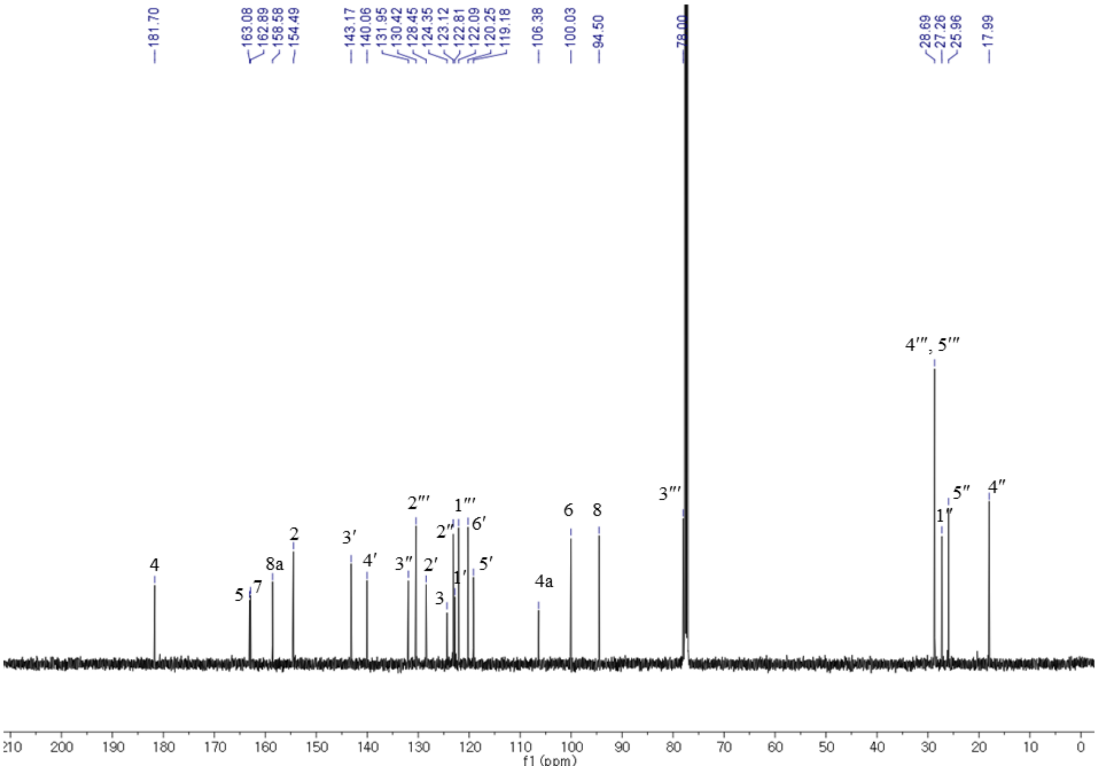

Figure S14. ^{13}C-NMR spectrum of compound **6** (125 MHz, $CDCl_3$)

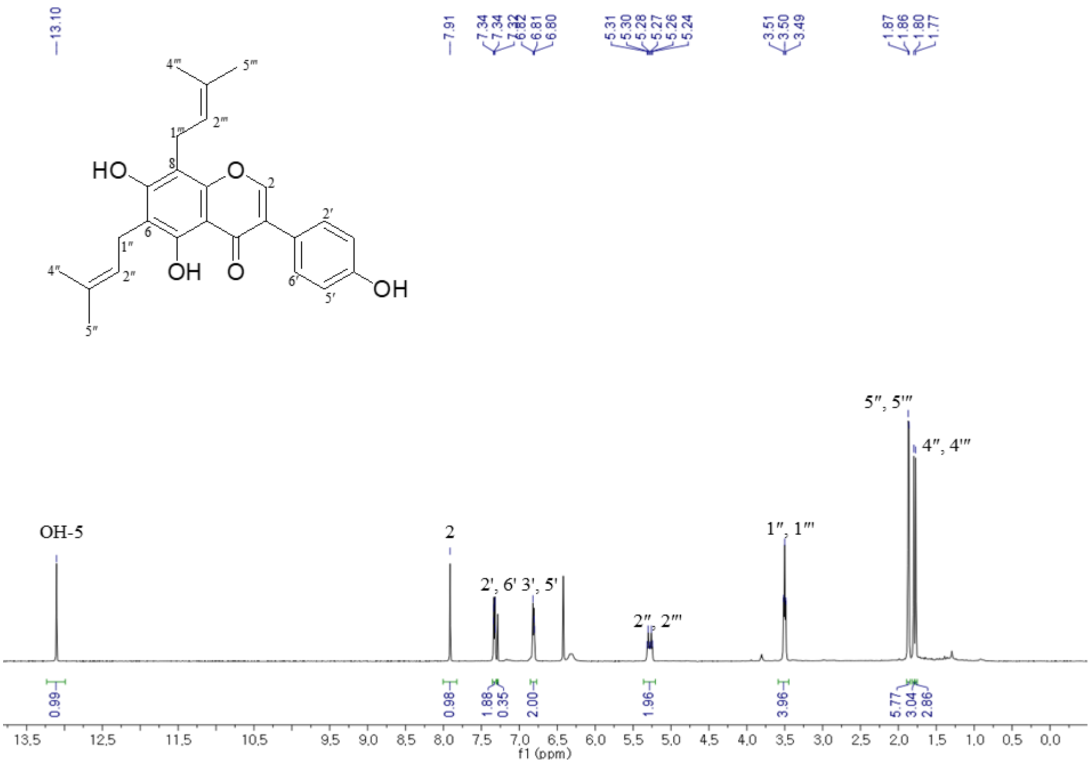

Figure S15. ^{1}H-NMR spectrum of compound **7** (500 MHz, $CDCl_3$)

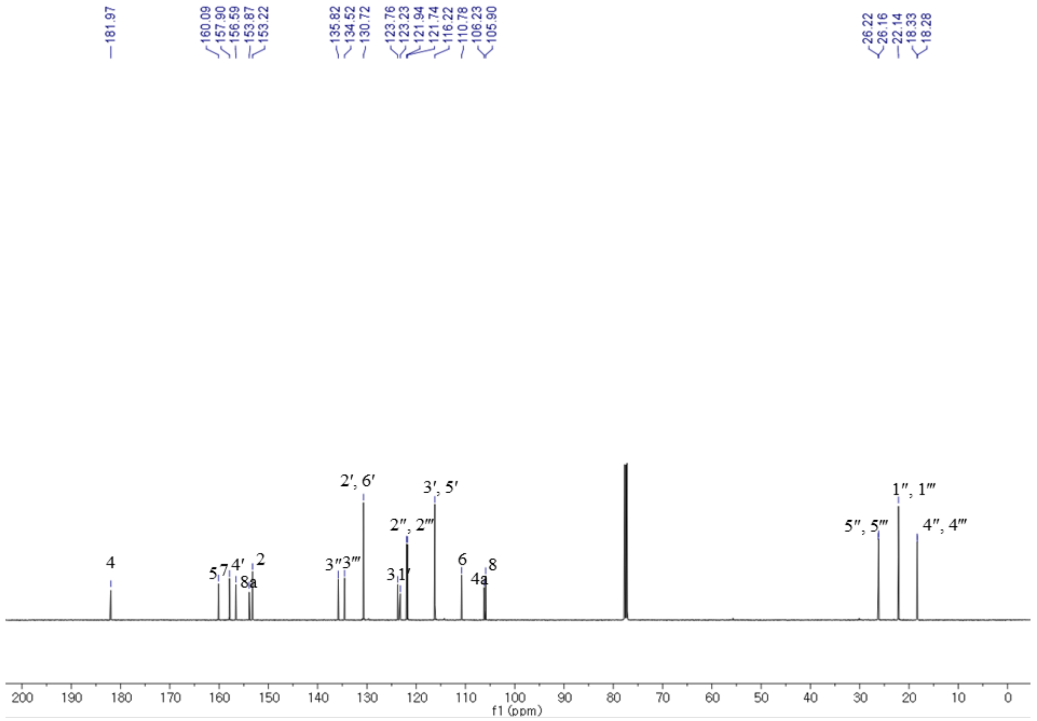

Figure S16. ^{13}C-NMR spectrum of compound **7** (125 MHz, $CDCl_3$)

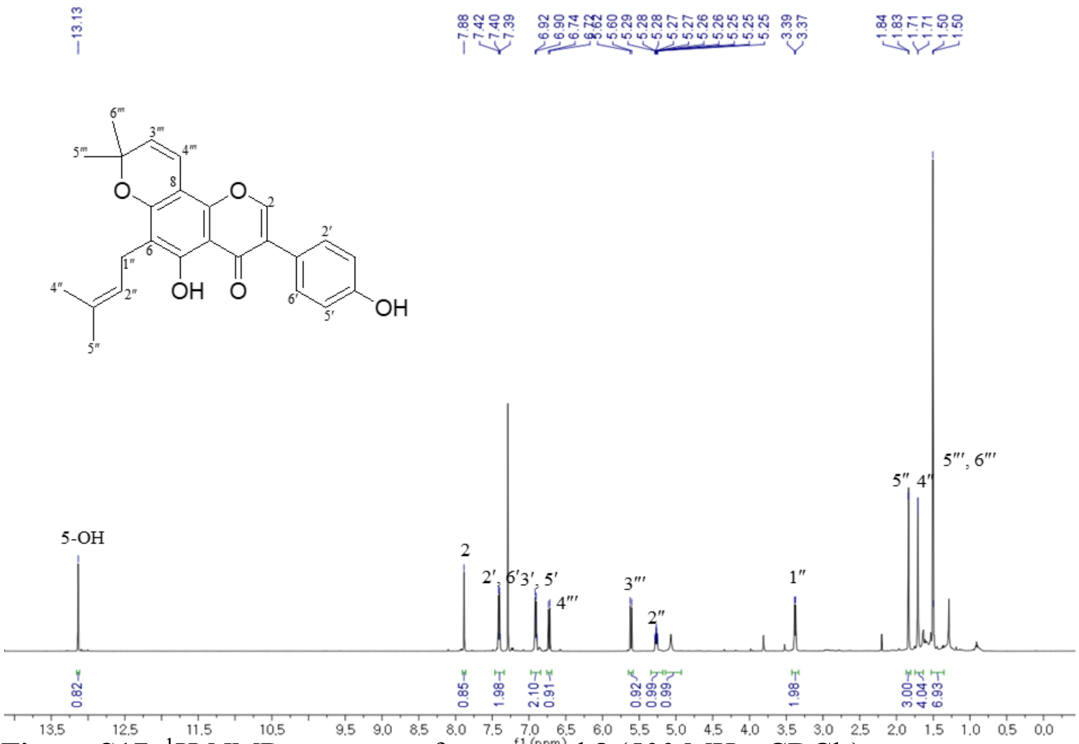

Figure S17. ^{1}H-NMR spectrum of compound **8** (500 MHz, $CDCl_3$)

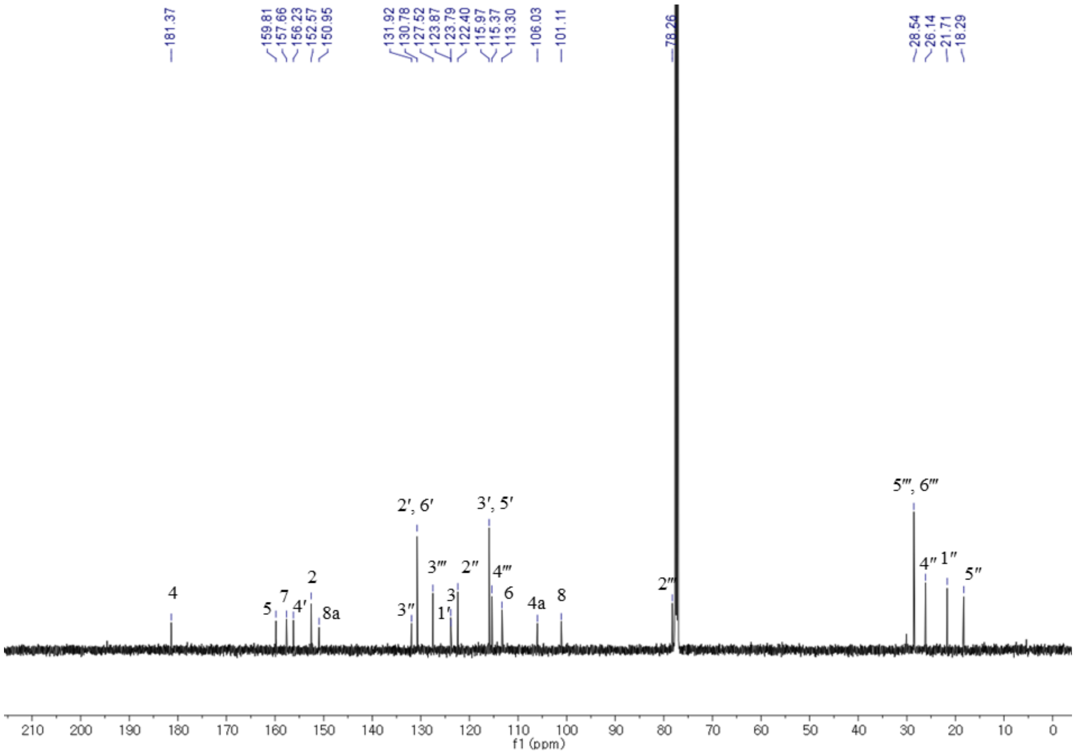

Figure S18. ^{13}C-NMR spectrum of compound **8** (125 MHz, $CDCl_3$)

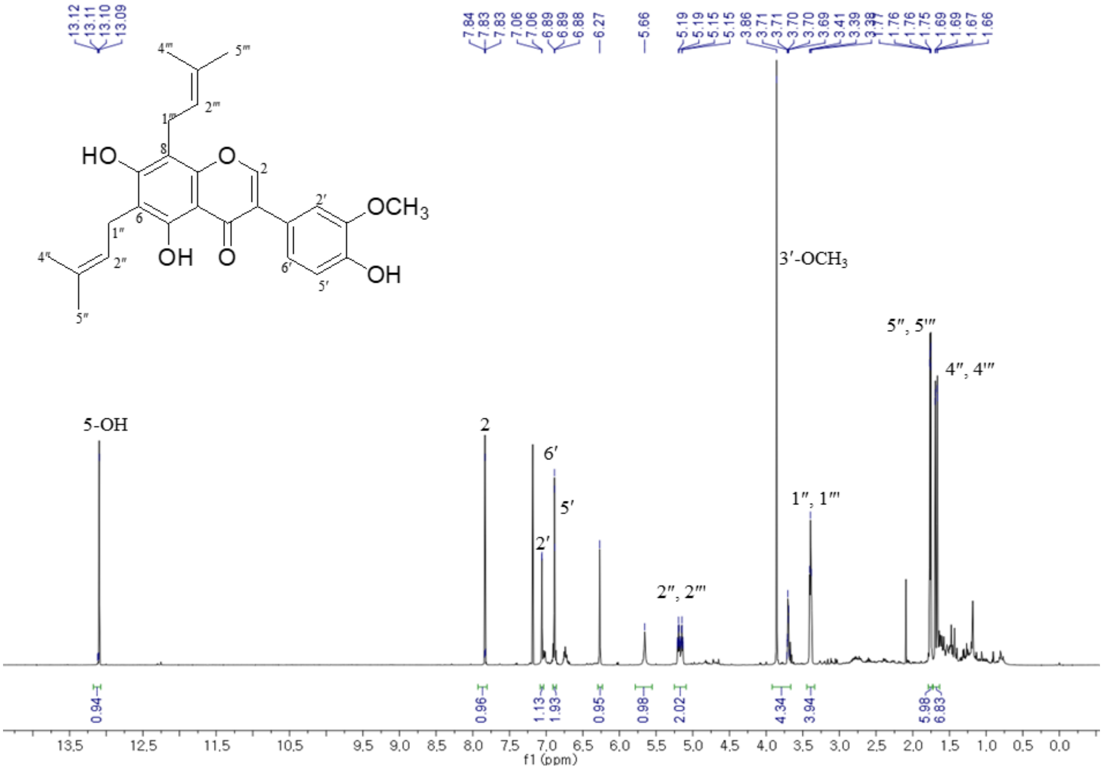

Figure S19. ^{1}H-NMR spectrum of compound **9** (500 MHz, $CDCl_3$)

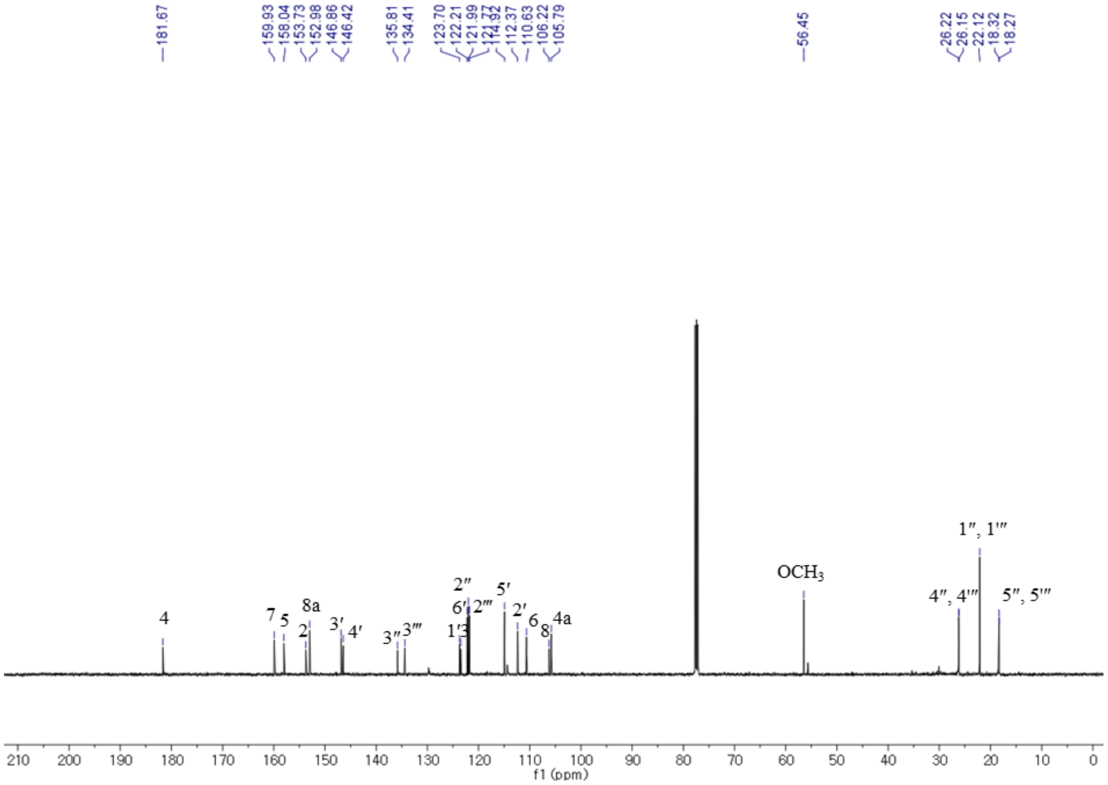

Figure S20. ^{13}C-NMR spectrum of compound **9** (125 MHz, $CDCl_3$)

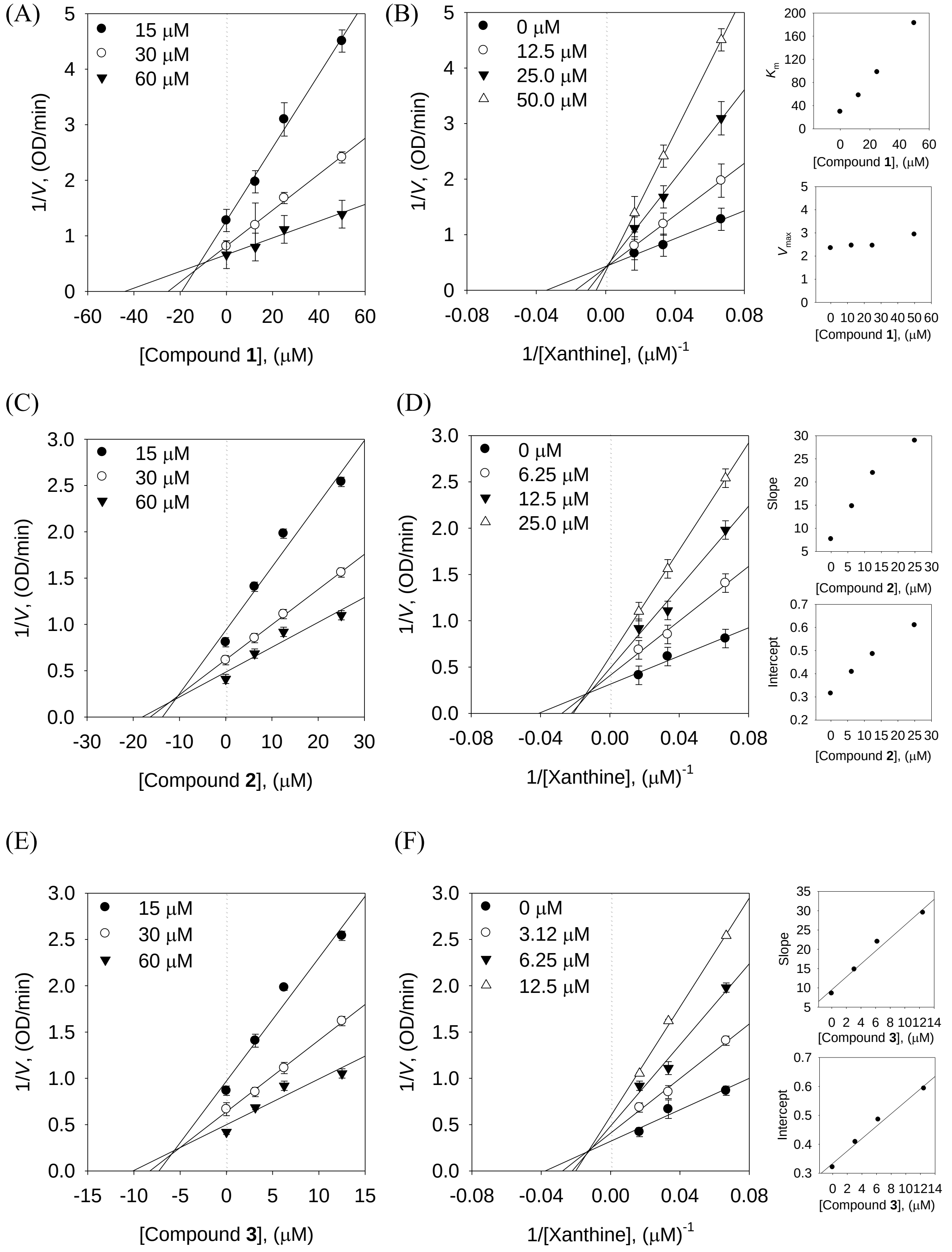

Figure S21. Lineweaver-Burk plots for the XO inhibition of compounds (A) **1**, (C) **2**, (E) **3**. Dixon plots for the XO inhibition of compounds (B) **1**, (D) **2**, (F) **3**.

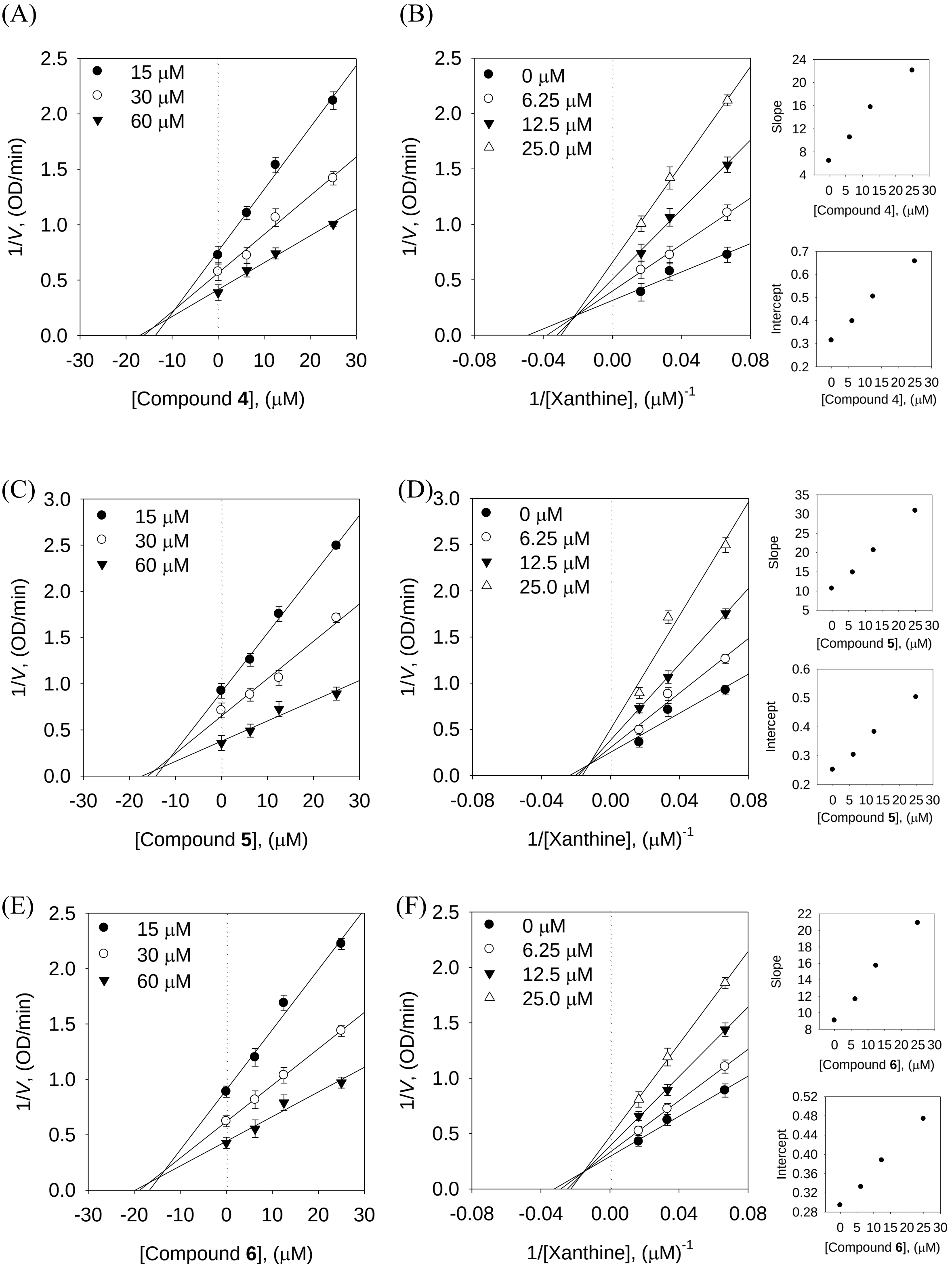

Figure S22. Lineweaver-Burk plots for the XO inhibition of compounds (A) **4**, (C) **5**, (E) **6**. Dixon plots for the XO inhibition of compounds (B) **4**, (D) **5**, (F) **6**.

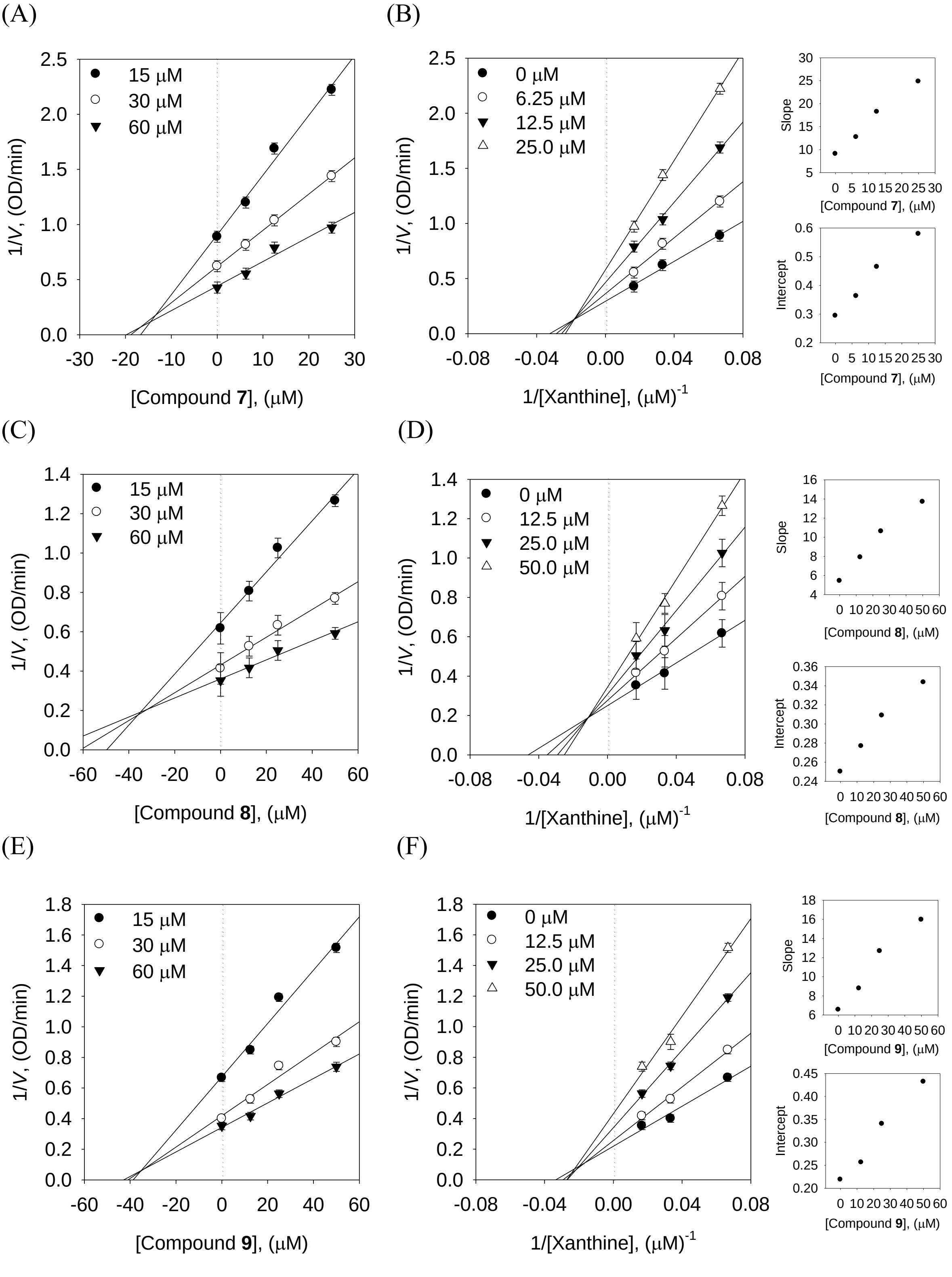

Figure S23. Lineweaver-Burk plots for the XO inhibition of compounds (A) **7**, (C) **8**, (E) **9**. Dixon plots for the XO inhibition of compounds (B) **7**, (D) **8**, (F) **9**.

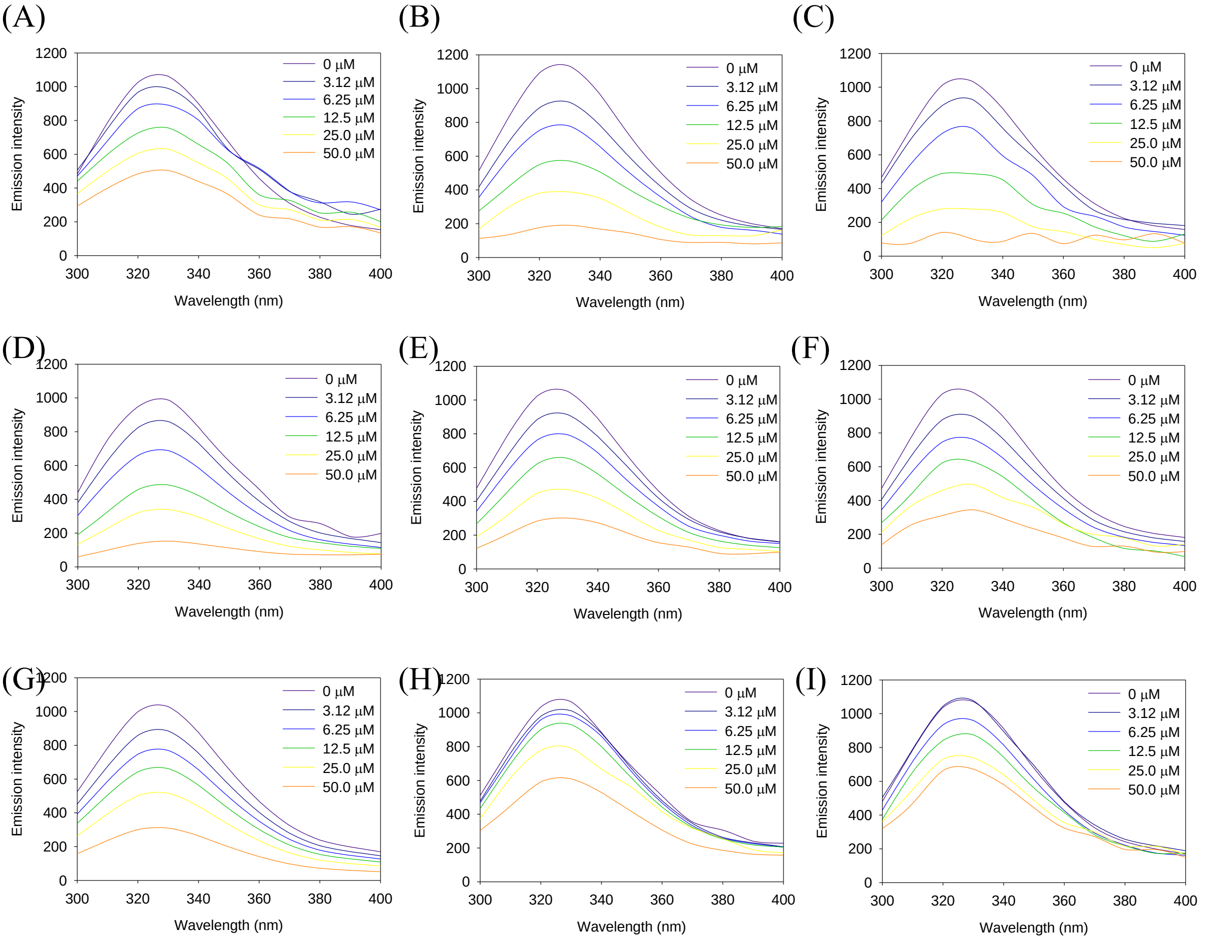

Figure S24. Binding affinity between XO and isoflavones (A) **1**, (B) **2**, (C) **3**, (D) **4**, (E) **5**, (F) **6**, (G) **7**, (H) **8**, and (I) **9**.

Table S1. Evaluation of Stern-Volmer constants depending on fluorescence quenching effects of xanthine oxidase inhibitors

Compounds	K_{SV} (x10^5 L·mol^{-1})	R^2	K_A(x10^6 L·mol^{-1})	n	R^2
**1**	0.1845	0.9944	0.5015	0.7268	0.9992
**2**	0.7755	0.9991	0.7544	1.0359	0.9984
**3**	1.2530	0.9998	0.8998	1.4392	0.9964
**4**	0.9497	0.9999	0.8896	1.4031	0.9961
**5**	0.5531	0.9999	0.7249	1.0018	0.9967
**6**	0.3645	0.9999	0.6240	0.8154	0.9983
**7**	0.4797	0.9914	0.6651	0.8836	0.9980
**8**	0.0987	0.9998	0.4667	0.6889	0.9987
**9**	0.0930	0.9804	0.2434	0.6736	0.9998

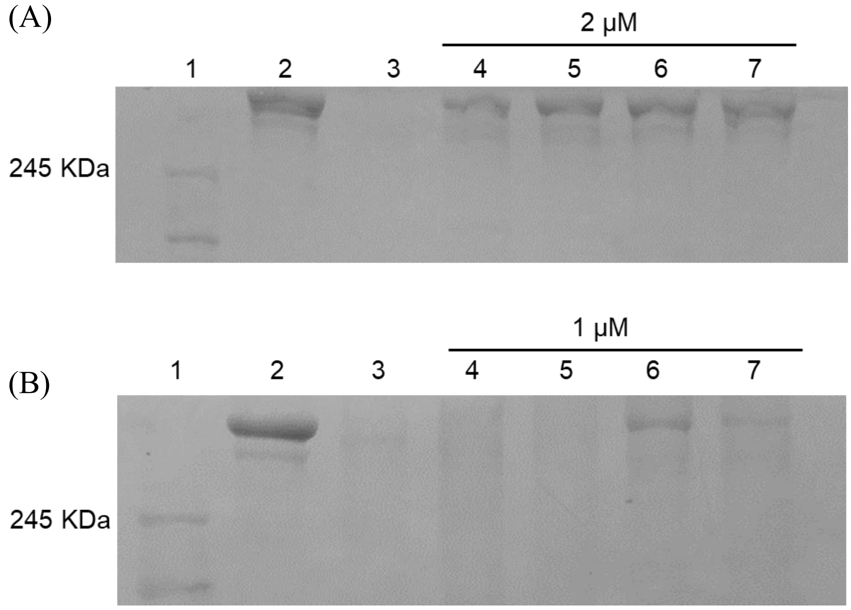

Figure S25. Effects of isoflavones (**2**, **3**, **4**, and **5**) on the ApoB-100 fragmentation. (A) Lane 1, marker; lane 2, native LDL; lane 3, ox-LDL; lane 4, **2** (2 μM); lane 5, **3** (2 μM); lane 6, **4** (2 μM); lane 7, **5** (2 μM). (B) Lane 1, marker; lane 2, native LDL; lane 3, ox-LDL; lane 4, **2** (1 μM); lane 5, **3** (1 μM); lane 6, **4** (1 μM); lane 7, **5** (1 μM).

Table S2. Characterization of isoflavones from methanol extract of *F. philippinensis* by UPLC-ESI-TOF-MS.

t_R (min)	Formula	Neutral mass (Da)	Observed neutral mass (Da)	Experimental *m/z* $[M+H]^+$	Fragmental ion *m/z*	Error (mDa)	Identification
3.51	$C_{15}H_{10}O_5$	270.0528	270.0528	271.06004			genistein (**1**)
4.49	$C_{25}H_{26}O_6$	422.1729	422.1733	423.18057	367, 311	+0.4	5,7,3',4'-tetrahydroxy-2',5'-di(3-methylbut-2-enyl)isoflavone (**4**)
4.76	$C_{25}H_{24}O_6$	420.1573	420.1582	421.16547	365, 283	+0.9	5, 7, 3'-trihydroxy-2'-(3-methylbut-2-enyl)-4',5'-(3,3-dimethylpyrano)isoflavone (**6**)
5.17	$C_{25}H_{26}O_6$	422.1729	422.1733	423.18057	367, 311	+0.4	6,8-diprenylorobol (**3**)
5.31	$C_{26}H_{28}O_6$	436.1886	436.1895	437.19678	381, 311	+0.9	flemingsin (**9**)
5.49	$C_{25}H_{26}O_5$	406.1780	406.1780	407.18525	351, 295		8-γ,γ-dimethylallylwighteone (**7**)
5.82	$C_{25}H_{24}O_6$	420.1573	420.1582	421.16548	365	+0.9	auriculasin (**2**)
6.14	$C_{25}H_{24}O_5$	404.1624	404.1633	405.17058	349	+0.9	osajin (**8**)
6.49	$C_{30}H_{32}O_6$	488.2199	488.2210	489.22831	433, 365	+1.1	flemiphilippinin A (**5**)

Sequence

Sequence status: Complete.
Sequence processing: The displayed sequence is further processed into a mature form.

P80457-1 [UniParc] FASTA Add to basket
« Hide

```
        10         20         30         40         50
MTADELVFFV NGKKVVEKNA DPETTLLAYL RRKLGLRGTK LGCGEGGCGA
        60         70         80         90        100
CTVMLSKYDR LQDKIIHFSA NACLAPICTL HHVAVTTVEG IGSTKTRLHP
       110        120        130        140        150
VQERIAKSHG SQCGFCTPGI VMSMYTLLRN QPEPTVEEIE DAFQGNLCRC
       160        170        180        190        200
TGYRPILQGF RTFAKNGGCC GGNGNNPNCC MNQKKDHTVT LSPSLFNPEE
       210        220        230        240        250
FMPLDPTQEP IFPPELLRLK DVPPKQLRFE GERVTWIQAS TLKELLDLKA
       260        270        280        290        300
QHPEAKLVVG NTEIGIEMKF KNQLFPMIIC PAWIPELNAV EHGPEGISFG
       310        320        330        340        350
AACALSSVEK TLLEAVAKLP TQKTEVFRGV LEQLRWFAGK QVKSVASLGG
       360        370        380        390        400
NIITASPISD LNPVFMASGT KLTIVSRGTR RTVPMDHTFF PSYRKTLLGP
       410        420        430        440        450
EEILLSIEIP YSREDEFFSA FKQASRREDD IAKVTCGMRV LFQPGSMQVK
       460        470        480        490        500
ELALCYGGMA DRTISALKTT QKQLSKFWNE KLLQDVCAGL AEELSLSPDA
       510        520        530        540        550
PGGMIEFRRT LTLSFFFKFY LTVLKKLGKD SKDKCGKLDP TYTSATLLFQ
       560        570        580        590        600
KDPPANIQLF QEVPNGQSKE DTVGRPLPHL AAAMQASGEA VYCDDIPRYE
       610        620        630        640        650
NELFLRLVTS TRAHAKIKSI DVSEAQKVPG FVCFLSADDI PGSNETGLFN
       660        670        680        690        700
DETVFAKDTV TCVGHIIGAV VADTPEHAER AAHVVKVTYE DLPAIITIED
       710        720        730        740        750
AIKNNSFYGS ELKIEKGDLK KGFSEADNVV SGELYIGGQD HFYLETHCTI
       760        770        780        790        800
AIPKGEEGEM ELFVSTQNAM KTQSFVAKML GVPVNRILVR VKRMGGGFGG
       810        820        830        840        850
KETRSTLVSV AVALAAYKTG HPVRCMLDRN EDMLITGGRH PFLARYKVGF
       860        870        880        890        900
MKTGTIVALE VDHYSNAGNS RDLSHSIMER ALFHMDNCYK IPNIRGTGRL
       910        920        930        940        950
CKTNLSSNTA FRGFGGPQAL FIAENWMSEV AVTCGLPAEE VRWKNMYKEG
       960        970        980        990       1000
DLTHFNQRLE GFSVPRCWDE CLKSSQYYAR KSEVDKFNKE NCWKKRGLCI
      1010       1020       1030       1040       1050
IPTKFGISFT VPFLNQAGAL IHVYTDGSVL VSHGGTEMGQ GLHTKMVQVA
      1060       1070       1080       1090       1100
SKALKIPISK IYISETSTNT VPNSSPTAAS VSTDIYGQAV YEACQTILKR
      1110       1120       1130       1140       1150
LEPFKKKNPD GSWEDWVMAA YQDRVSLSTT GFYRTPNLGY SFETNSGNAF
      1160       1170       1180       1190       1200
HYFTYGVACS EVEIDCLTGD HKNLRTDIVM DVGSSLNPAI DIGQVEGAFV
      1210       1220       1230       1240       1250
QGLGLFTLEE LHYSPEGSLH TRGPSTYKIP AFGSIPTEFR VSLLRDCPNK
      1260       1270       1280       1290       1300
KAIYASKAVG EPPLFLGASV FFAIKDAIRA ARAQHTNNNT KELFRLDSPA
      1310       1320       1330
TPEKIRNACV DKFTTLCVTG APGNCKPWSL RV
```

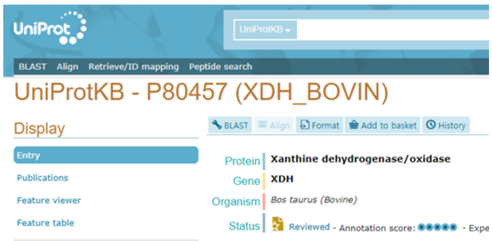

Figure S26. Amino acids sequence of xanthine oxidase from bovine milk.
